# Language Outcomes in Cochlear Implanted Children with White Matter Disturbances

**DOI:** 10.22038/ijorl.2021.48909.2621

**Published:** 2021-09

**Authors:** Anuradha Sharma, Naresh Panda, Sanjay Munjal

**Affiliations:** 1 *Department of Otolaryngology, New OPD, PGIMER, Sector 12, Chandigarh, India.*; 2 *Department of Otolaryngology, Post Graduate Institute of Medical Education and Research, Chandigarh, India.*

**Keywords:** Auditory verbal therapy, Cochlear Implant, Leukodystrophy, White matter disturbances, Severe to profound hearing loss

## Abstract

**Introduction:**

The present study reviews our experience with children with white matter disturbances and the benefits they get from rehabilitation post cochlear implantation.

**Materials and Methods:**

It is a retrospective cohort study of 7 cochlear implanted children with white matter disturbances. Preoperatively all the subjects had undergone a complete Audiological test battery for confirmation of hearing thresholds. Post assessment, a digital hearing aid trial was followed by three months’ therapy. Unilateral cochlear implant surgery and monitored auditory-verbal therapy sessions were the next line of treatment for at least one year. The therapist regularly monitored hearing and communication outcomes on an Auditory verbal ongoing scale, revised CAP, MAIS, word, and sentence discrimination scores.

**Results:**

The age range of Implantation was between 48 to 60 months. 5 out of 7 participants showed remarkable improvement with regular therapy. Their Meaningful Auditory Integration Scale (MAIS) scores were greater than 35 indicating good auditory integration and Categories of Auditory Performance (CAP) revealed scores of even 9 and higher indicating good telephone conversation. Speech Intelligibility Rating (SIR) showed a rating of 4 meaning thereby that an unfamiliar Listener could understand Speech without additional cues. However, all of them reported difficulty perceiving speech in noisy environments. Two cochlear implantees needed speech reading cues in conjunction with the audition.

**Conclusion:**

Our experience with cochlear Implantation in children with white matter abnormalities has been positive and satisfactory. The presence of white matter abnormalities on MRI should not be a contraindication for Implantation. Successful outcomes can be expected with regular and dedicated auditory-verbal therapy sessions.

## Introduction

Hearing loss is an invisible impairment and constitutes 5.3% of the world's population. The National Sample Survey (NSS,2002) has reported hearing impairment as the 2^nd^ most prevalent disability in India (1). Cochlear Implantation is considered the most effective tool for the management of severe to profound sensorineural hearing loss. Despite advancements in technology, it is also well known that multiple intrinsic and extrinsic factors impact hearing and language outcomes (2-3). A significant number of audiological and extra audiological variables like the onset of hearing loss, residual hearing, presence of coexisting disabilities, malformations of the ear, brain anomalies, socioeconomic status and familial environment have an immense impact on speech and language outcomes post cochlear Implantation (4-6). With increased heterogeneity due to the variables mentioned above, it becomes a challenging task to predict the outcomes of post cochlear implant surgery (7-9). Periventricular white matter injury is the most potent injury leading to significant long term neurologic deficits in an infant (10). 

White matter disturbances have been associated with gait and postural defects, ophthalmological disorders, learning problems and attention abnormalities (11). The disease could be progressive in some, especially in children with late-onset lesions and non-progressive in others with asymmetric white matter lesions (12). 

A cochlear implant is an invasive and expensive procedure; identification of appropriate candidacy is the primary goal. Successful outcomes with cochlear Implantation are comparable to regular hearing counterparts in Hearing, Speech, and Language performance (13). Early intervention with a cochlear implant becomes the prime mode of intervention in children who do not gain adequate benefit from traditional hearing aids (14). It allows the optimal development of the child's auditory, cognitive, and linguistic development. To the best of our knowledge, there is a shortage of literature analyzing the long-term speech and language outcomes (8-14years post switch on) in the pediatric cochlear implantees having white matter disturbances. Therefore, the study analyzed the long-term effects (8-14 years) of cochlear implantation on language development in children with white matter disturbances. 

## Materials and Methods

This study retrospectively analyzed data from cochlear implanted subjects with white matter disturbances. The study sample consisted of prelingual cochlear implantees who had attended regular auditory-verbal therapy at a tertiary care hospital. All the subjects had undergone free field audiometry, Immittance Audiometry, Auditory Brainstem Response (ABR), and Otoacoustic Emissions (OAE) to confirm severe to profound hearing loss. The study sample comprises five males and two females, age range 7-17 years (Table.1).

**Table 1:** Pre implant demographics of patients

**Implantee** **(case number)**	**Present age**	**PTA/BOA**	**ABR**	**Age at which Hearing aid fitting**	**Duration of hearing aid use**	**Duration of Speech and language therapy with the hearing aid**
		Right left (dBHL) (dBHL)				
1	16yr/M	>117 >110	B/L profound loss	2 year	1 year	1 year
2	11yr/M	>110 >110	B/L severe loss	2.5 yr	5 month	5 month
3	8 yr /M	>100 >100	B/L profound loss	6 month	1.5 yr	1 yr
4	7yr/F	>105 >120	B/L profound loss	7 month	8 month	8 month
5	17/M	>100 >100	B/L profound loss	3 yr	1yr	6 month
6	17/F	>110 >110	B/L profound loss	3 yr	6 month	6 month
7	11/M	>100 >100	B/L Severe loss	4 yr	6 month	6 month

A thorough case history was acquired that included patient demographics, chronological age, gender, the age at which fitted with a hearing aid, the age of cochlear Implantation and pre-peri- and post-natal medical history. All the subjects had undergone regular Speech and language therapy for six months to 1 year with hearing aids before Implantation.

A retrospective chart review showed that a detailed pediatric and neurologic examination had been conducted before Implantation. None of the subjects had any neurologic deficits other than white matter changes in the brain. The patients with features of leukodystrophy on MRI were assessed by the pediatric Neurologists and Intensivists. All the subjects reporting white matter changes were classified into three categories: mild – showing periventricular temporal, anterior and posterior horn or body involvement, moderate – showing two or three lesions and severe – showing 3 or more lesions. Most of them had associated bilateral profound hearing loss for which they were being considered for Cochlear Implantation. Additionally, some of them had features of attention deficit hyperactivity disorder. One of the subjects had motor difficulty, one patient had features of mild athetoid Cerebral palsy and developmental delay. However, none of them had any seizure disorder. 

Imaging data

**Fig 1:**
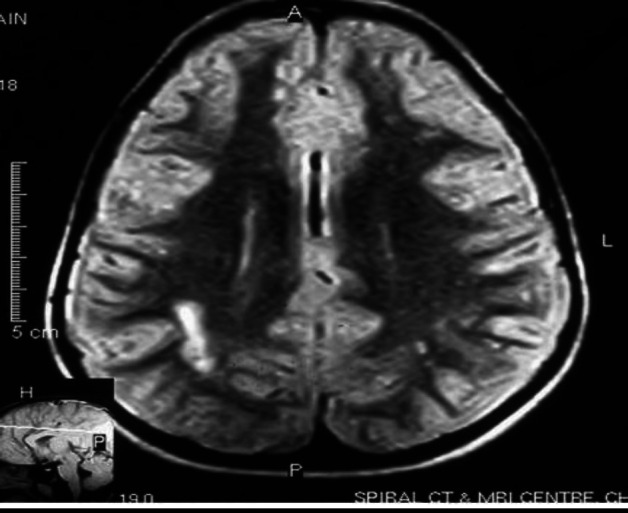
*Parieto occipital -Middle Cerebral and posterior cerebral area showing white matter changes*

**Fig 2:**
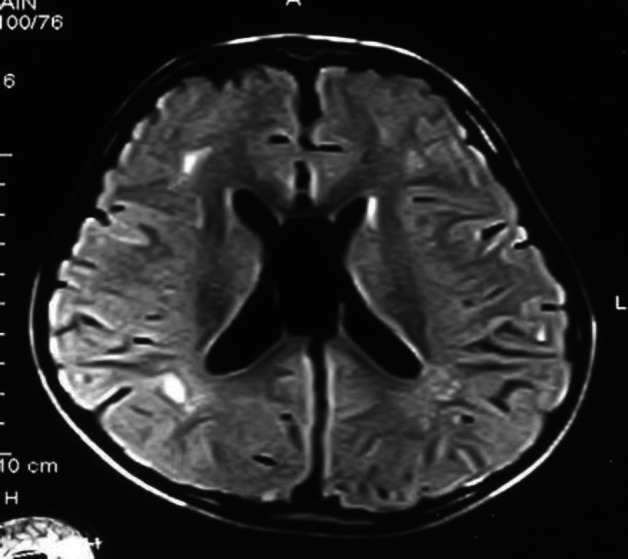
*Periventricular focal gliosis in parieto occipital area in white matter*

High-resolution HRCT and MRI of temporal bone and brain had confirmed the presence of white matter disturbances in all seven subjects. MRI scans of two patients are shown below for illustration (figure1, 2, and 3) that show focal or diffuse lesions within the white matter.

Speech and language assessment was conducted through various scales and tests, viz; Auditory verbal ongoing scale (AVOS), categories of auditory performance (CAP), Meaningful auditory integration scale (MAIS), Speech intelligibility rating scale (SIR), and Speech discrimination ability (using a Punjabi PB word list)

## Results

The purpose of presenting this case study was to characterize the long-term speech and language outcomes in implantees with white matter disturbances.

Case 1 

The subject is a 16-year-old male, implanted on the right ear at the age of 4. He was diagnosed at 2years with a bilateral profound sensorineural loss with the absence of ABR waves at 99dBnHL and absent OAEs. Digital hearing aids were accepted quickly by the subject, and his parents regularly attended therapy sessions. Due to the limited benefit even after three months of regular hearing aid usage, the child was worked up for Cochlear Implantation. 

Detailed radiological examination revealed mild to moderate peri-ventricular and deep white matter changes. The psychological evaluation revealed above average intellectual functioning with no behavioral issues. The radiological study further showed that the cochlear anatomy was normal in structure. He was implanted with CI 24 RE (ST) cochlear implant at 4 years of age using a round window approach with more than 270-degree insertion. The post-surgery subject underwent intensive Speech and language therapy for two years. His mother attended two sessions (each lasting for 45 minutes) per week for the first six months and one session per week after that. The mother of the child was very enthusiastic and she actively participated during the sessions. She carried out all the home training tasks religiously. The subject attained complex language and pragmatic skills with a sprint processor (Cochlear), which he has been satisfactorily wearing for the past 12 years. He demonstrates excellent listening skills, such as open set listening, auditory memory and auditory sequencing. He listens to music, talks on the telephone, and his speech intelligibility is good. However, he reports intermittent difficulty articulating a few consonants, especially the blends (Table 2-4).

**Table 2:** Post implant demographics

**Implantee** **Case number**	**Associated problems**	**Age at which implanted**	**Duration of implant usage in years**	**Implant type**	**Type of Speech Processor**	**Duration of AVT**
1	NIL	4 yr	12yr	Nucleus CI 24 RE (ST)	Sprint (cochlear nucleus )	2yr
2	NIL	5 yr	6yr	Nucleus CI 24 RE (ST)	Freedom (cochlear nucleus)	2yr
3	Nil	2yr	6yr	Nucleus CI 24 RE (ST)	Freedom (cochlear nucleus)	1yr
4	Motor difficulties	1.5 yr	5.5yr	Nucleus CI 24 RE (CA)	CP810( cochlear nucleus	2yr
5	Cerebral palsy	5 yr	12yr	Nucleus CI 24 RE (ST)	Sprint (cochlear nucleus	1yr
6	Dental anomalies	5yr	12yr	Nucleus CI 24 RE (ST)	Sprint (cochlear nucleus), Currently using CP 810	2yr
7	Attention deficit hyperactivity disorder	5 yr	6yr	Nucleus CI 24 RE (ST)	Freedom (cochlear nucleus)	1yr

**Table 3:** Cochlear implantees performance scores

**Implantee** **Case number**	**Aided auditory thresholds (Average of 500,1KHz,2KHz )**	**Implant age**	**Auditory verbal ongoing scale** **Audition**	**Auditory verbal ongoing scale** **Language**	**Auditory verbal ongoing scale** **Speech**	**Auditory verbal ongoing scale** **communication**	**Revised CAP score**	**MAIS**
1	30dB	12yr	Consistent on instruction and stories	Acquired all the sub parts	Only Acquisition of blends is inconsistent	Maintains topic upto 3 turns	11	38
2	25dB	6yr	Open set stage	uses verbs pronouns and prepositions	All vowels and consonants consistent	Repair strategies inconsistent	8	38
3	20dB	6yr	Open set stage	Acquired all sub parts	All vowels and consonants consistent	Maintains topic 3 turns	11	38
4	35dB	5.5yr	Open set stage	Inconsistent usage	Blends and affricates inconsistent	Repair strategies inconsistent	9	25
5	15dB	12yr	Open set stage	Inconsistent in auxiliary questions/articles	Misarticulated speech	Repair strategies inconsistent	9	33
6	15dB	12yr	Words in phrases	Only nouns	Very few consonants and vowels	Communication strategies inconsistent	5	17
7	30dB	6yr	Words in phrases	Nouns and verbs	Very few consonants and vowels	Communication strategies at beginning stage	6	16

**Table 4:** Cochlear implantees word and sentence discrimination scores

**Implantee case number**	**Word discrimination score**	**Sentence discrimination score**	**Word discrimination score in noise**	**Sentence discrimination score in noise**	**Speech Intelligibility** **Rating**
1	90%	80%	75%	60%	5
2	80%	60%	60%	50%	4
3	90%	85%	75%	70%	5
4	80%	70%	60%	50%	4
5	70%	60%	50%	30%	3
6	7%	3%	0%	0%	2
7	9%	5%	5%	3%	2
					

**Case 2**

The subject reported to the tertiary care hospital at the age of 4.5 years, with severe bilateral loss. He was using a digital hearing aid and had attended regular speech therapy for five months at a private clinic, but with a limited benefit. The child was already using gestures along with a few words for communication needs. The workup for cochlear Implantation included a thorough medical and radiological examination which confirmed the absence of inner ear malformations. Neurological examination findings confirmed normal central nervous system functioning. The preoperative psychological assessment showed average intelligence with no significant behavioral concerns. MRI showed increased T2 signals in the medial temporal lobe region and changes in the right frontal lobe. At 5years of age, a round window surgical technique was employed to achieve complete electrode insertion of Nucleus CI 24 RE (ST). The auditory-verbal therapy continued for 2years post-implantation with a freedom processor. The most challenging part was to reduce reliance on gestures for communication and increase auditory comprehension. The parents were very much inclined towards speech rehabilitation and attended speech therapy at a center near their home as well as at the rehabilitation unit of the tertiary care center. The subject presented with a few behavioral issues in the first few sessions of auditory verbal therapy. Time out, along with contingent reinforcement techniques helped to achieve the desired goals at a regular pace. The subject can currently discriminate words and sentences in noise at 60% and 50%, respectively. Connected Speech is intelligible to the Listener with a little concentration (Table 2-4).

*Case 3*

The subject was the younger brother of the case 2, so he reported very young (at the age of 1 year). He also presented with bilateral profound sensorineural hearing loss. Despite being fitted with digital aids at 1.5 years of age, the subject could attain no optimum benefit in auditory, speech and language domains. The cochlear implant surgery was expedited and a detailed neurological, pediatric, radiological, and psychological evaluation followed thereon. After attaining a neurological and pediatric clearance, radiological examination results showed no dysplasia of the Inner ear canal. MRI showed scattered white matter changes indicative of underlying leukodystrophy. The psychologist's observation indicated no cognitive issues. At two years the subject was implanted with Nucleus CI 24 RE (ST) without any complications. The electrode insertion was complete with the round window technique and the Neural Response Telemetry could be obtained on all the electrodes. Post-switch-on the verbal interactions between the siblings started increasing drastically. However, being the youngest child of the family, the subject had many issues relating to behavior and discipline. He was not cooperative for auditory verbal therapy sessions during initial sittings. Gradually, with a cumulative effort of parents as well as grandparents followed by strenuous therapy, the child showed considerable progress. The subject's mother had attained a great deal of confidence post AVT sessions of the elder sibling. She very keenly took the initiative and carried out many group tasks at home to facilitate speech and language development.

As a result, the subject has attained audition and language goals much faster (table 2-4). The subject has developed excellent conversational skills in a variety of contexts and can predict what will happen. An unknown listener can understand his connected Speech in all contexts. His Auditory, Speech and language levels are elaborated in Tables 2, 3, and 4.

**Case 4**

The Pediatrician referred the child with suspected hearing loss and associated mild motor and visual difficulties at eight months for audition and language goals to the tertiary care. The wave V could not be identified on auditory brainstem response even at 99dBnHL and OAEs were also absent. The audiologist fitted him with Naida Q 30 Phonak hearing aids in both ears. The parents were very motivated and regularly brought the child for therapy. However, consistent responses could be observed during auditory training sessions only for low-frequency sounds like that of the drum. She did not respond even to name call after four months of regular therapy. At 1.5 years of age, the cochlear implant team reevaluated the subject's performance and planned for cochlear implant surgery. The neurologist and pediatrician did their preliminary workup, and no contraindications for surgery were reported. The radiological investigations showed white matter changes in Frontal –anterior and middle cerebral, parieto occipital, and posterior cerebral areas. The psychologist mentioned that the child was shy and took time to build rapport. However, the social quotient (SQ) was age-appropriate. Nucleus CI 24 RE (CA) with a freedom processor was implanted at 1.5 years. The round window surgical technique led to an uneventful surgery, and Intraoperative NRT confirmed the same. She slowly adjusted to her implanted device.

Regular mapping sessions ensured a full range of hearing. Parents shifted their base near tertiary care hospital for almost six months for intensive auditory-verbal therapy sessions. The mother worked tirelessly with the daughter, using various auditory-verbal strategies like acoustic highlighting and focused attention to develop listening skills. She is now six years of age; her mean length of utterance is 4-5 words and articulatory errors are frequent. Speech intelligibility is fair; the Listener can comprehend with few repetitions (Table 2-4). She is a listening, thinking and talking child. However, she is still facing learning problems at school and her grades are compromised.

*Case 5*

The subject is a 17-year-old female. She was diagnosed with bilateral sensorineural hearing loss at the chronological age of 4. She was fitted with digital aids (Danavox aid) and she attended regular therapy for six months. However, even after intensive auditory training with hearing aids, she could only detect the low-frequency Ling sounds at a distance of five feet. Her responses to even simple verbal instructions were minimal. She had associated Gait disturbances (mild athetoid movements) and orthodontic anomalies as assessed by the pediatrician. MRI of the brain showed bilateral nearly symmetrical white matter T2 and fluid-attenuated inversion recovery (FLAIR) hyper intensities with diffuse involvement. The radiologist's reports did not indicate any cochlear deformities or underlying diseases. The psychological assessment did not suspect any intellectual disability. At the age of 5, she was implanted with nucleus CI 24 RE (ST), using a round window surgical technique. All the electrodes were active, and within weeks, the cochlear implant was used for all waking hours. Her aided audiogram with a sprint processor was within speech banana, post one month of the switch-on. She continued auditory-verbal therapy for 1.5 years; however, her verbal comprehension and expression were limited despite excellent aided thresholds. Her mother was highly motivated and would continuously expand the child's language through meaningful interaction and encouraged her to progress from simple to complex sentences. The therapist incorporated many techniques during auditory-verbal therapy sessions like auditory bombardment, imitation, expansion, and adding meaning to sound. The subject could identify items from closed set tasks but demonstrated decreased performance in the open set. Presently the child is using both oral and manual modes for communication (Table 2-4). She is processing simple language through listening. She is also pursuing a vocational course after class 10.

**Case 6**

The subject was the twin brother of case 5, referred by the pediatric neurology department at 4 years of age with suspected hearing loss. He also presented with orthodontic and facial deformities. The subject's medical records were summarized as having white matter changes within the right parietal region suggestive of underlying leukodystrophy. The parents did not proceed with genetic testing and were keen on initiating the management. Bilateral profound sensorineural hearing loss was confirmed after detailed audiological assessment through Auditory brainstem response, otoacoustic emissions, pure tone audiometry, followed by Immittance audiometry etc. He did not benefit from digital and analog hearing aids. Hence, he was referred for cochlear implant surgery. He was fitted with binaural strong class hearing aids and he underwent language therapy during the preimplant candidacy assessments. By this time, the child had adopted lip-reading skills and manual signs as a mode of communication. Preimplant radiological evaluation ruled out any other brain or cochlear anomalies. At the chronological age of 5, Implantation was completed with Nucleus CI 24 RE (ST). The surgeon used round window insertion with a soft surgical approach. Sprint processor gave him excellent access to verbal stimuli in an extremely stimulating and language enriched environment. The therapist gave them a family-based program where the emphasis was on natural child-centered communication. The parents trained subjects 5 and 6 (twins) for 3-4 hours per day in natural settings. Partnership with professionals and his immediate and extended family's keen involvement fostered the subject's way towards the processing of even complex spoken language. He is currently 17 years and using an N6 speech processor. Speech is intelligible with a little effort, inconsistent use of grammatical markers and inappropriate repair strategies (Table 2-4). He occasionally faces difficulty understanding descriptive sentences in the environment of background noise and supplements it with Speech reading cues. Vocationally, he is pursuing a diploma course in computer programming.

**Case 7**

The subject was referred from the psychiatry department with mild global developmental delay, attention deficit hyperactivity disorder and inability to speak. His mother was also undergoing psychiatric treatment for depression. There was no other history of seizures, impaired vision, night blindness, or ataxia. Magnetic resonance imaging (MRI) of the brain indicated global hypomyelination. However, the brainstem and basal ganglia were normal. Pure tone and free field assessments were not conclusive about his hearing sensitivity. 

Objective tests viz (Brainstem evoked response audiometry, Otoacoustic emissions, Immitamce audiometry, and Auditory steady-state response) confirmed the diagnosis of bilateral severe to profound hearing loss. During the hearing aid fitting sessions, optimum responses were not observable with any analog or digital aid. Hence, the subject had been enrolled for diagnostic therapy sessions with digital hearing aids. He would frequently throw his hearing aids, and his parents had to shell out a lot of money to repair and maintain the aids. The psychiatrist's opinion helped to manage ADHD (Methylphenidate was prescribed). At the age of 5, a senior surgeon carried out a successful cochlear implant surgery with Nucleus CI 24 RE (ST) using a round window technique. Intraoperative NRT documented on electrodes 1-18 only. Following "switch on," frequent mapping sessions were required to establish conditioned MAPs. Only the father of the subject attended regular sessions and was highly cooperative. The child accepted the cochlear implant and wore it for approximately 5-6 hours a day. He started comprehending 2-3 word meaningful verbal commands and expressed himself through a few words and phrases along with rudimentary gestures (Table 2-4). However, even after one year of auditory verbal therapy, the adequate benefit could not be achieved. He is presently using both oral and manual modes of communication.

## Discussion

This study is novel in elaborating long-term speech and language outcomes in cochlear implantees with white matter changes. The present research typically evidenced satisfactory results (71.42% subjects), except for a few cases. There is a paucity of literature on long-term outcomes with cochlear implants in white matter disturbances. Most researchers have emphasized cochlear implants' role in malformed ears and compared them with deaf counterparts (15). Nevertheless, Trimble et al.(2008) reported 70% of their subjects with cochlear implants presented with white matter abnormalities, had a history of hypoxic insults, infection, ischemia, and prematurity (16).

Perlman JM (1998) commented that white matter changes may have an adverse impact on neurodevelopmental outcomes and may act as deterrents in managing cochlear implant subjects (10). Additionally, Luthra S. et al. also explained that leukodystrophies might interfere with expectations post–CI and indicate an Auditory processing disorder (17). 

The findings of these studies did not agree with those of ours, as 5 out of 7 cases showed significant improvement. MAIS scores with > 35 (showing good auditory integration) and CAP score of >9 (good perception on telephone conversations) implied optimum aural skills. Speech intelligibility rating (SIR) of > 4(indicative of an unfamiliar listener could understand Speech without additional cues) in these five subjects indicated reasonably good outcomes. Furthermore, they also demonstrated satisfactory word and sentence discrimination scores in quiet settings.

Busi et al. studied the outcome in subjects with brain anomalies and subjects with both inner ear malformations and brain abnormalities. They reported significant differences after 2-3 year follow-up in favor of subjects having only brain abnormalities. The subjects with brain anomalies reportedly had better performances (15).

Researchers have also emphasized significant differences in long term outcome scores after cochlear implantation in subjects with brain anomalies, implanted at older than 3 years of age (15). 

Robbins and colleagues observed that IT-MAIS results were favorable for children under 19 months of age with comorbidities (18). Gears (2004) has also supported his findings that a critical period of development (first two years of life) gives an added advantage to the implanted children (19). These results further support our findings that assessment and management precocity are essential predictor tools even for children with white matter disturbances. Subjects with white matter disturbances should be considered suitable candidates for Cochlear Implantation. Furthermore, the results of the current study also highlighted that children having associated problems and implanted after 3-4 years of chronological age may need lip-reading cues in conjunction with auditory cues for comprehension. Brain imaging studies like MRI might aid in appropriate counseling and appropriate expectations post-implantation from children with white matter disturbances/ leukodystrophy. 

Despite having limitations, i.e., retrospective case study and small sample size, our study provides positive evidence towards cochlear Implantation in white matter disturbances. 

Our results reflect an assessment of the extensive case series and support the earlier notion that Cochlear Implant surgery is a successful intervention technique with favorable long-term benefits in subjects with white matter abnormalities.

## Conclusion

Periventricular white matter injury remains a significant problem in almost 15-20% of infants. The current research reported positive outcomes of cochlear implanted children with white matter abnormalities. We believe that an experienced team of professionals and an early intervention would be the two critical factors for attaining successful implantation outcomes. Therefore, children showing demyelination on MRI are potential candidates for cochlear Implantation.
